# A Clickable Oxysterol Photolabel Retains NMDA Receptor Activity and Accumulates in Neurons

**DOI:** 10.3389/fnins.2018.00923

**Published:** 2018-12-06

**Authors:** Daniel M. Chen, Luke Ziolkowski, Ann Benz, Mingxing Qian, Charles F. Zorumski, Douglas F. Covey, Steven Mennerick

**Affiliations:** ^1^Department of Psychiatry, Washington University, St. Louis, MO, United States; ^2^Taylor Family Institute for Innovative Psychiatric Research, St. Louis, MO, United States; ^3^Department of Developmental Biology, Washington University, St. Louis MO, United States

**Keywords:** NMDA receptor, oxysterol, photolabel, click chemistry, modulation

## Abstract

Oxysterol analogs that modulate NMDA receptor function are candidates for therapeutic development to treat neuropsychiatric disorders. However, the cellular actions of these compounds are still unclear. For instance, how these compounds are compartmentalized or trafficked in neurons is unknown. In this study, we utilized a chemical biology approach combining photolabeling and click chemistry. We introduce a biologically active oxysterol analog that contains: (1) a diazirine group, allowing for the permanent labeling of cellular targets, and (2) an alkyne group, allowing for subsequent *in situ* visualization using Cu^2+^ catalyzed cycloaddition of an azide-conjugated fluorophore. The physiological properties of this analog at NMDA receptors resemble those of other oxysterols, including occlusion with other oxysterol-like compounds. Fluorescent imaging reveals that the analog accumulates diffusely in the cytoplasm of neurons through an energy-independent mechanism. Overall, this work introduces a novel chemical biology approach to investigate oxysterol actions and introduces a tool useful for further cell biological and biochemical studies of oxysterols.

## Introduction

N-methyl-D-aspartate receptors (NMDARs) are glutamate-gated ion channels that play important roles in neurobiological function. Normal NMDAR function is associated with synaptic plasticity important for encoding new memories, but abnormal NMDAR function is implicated in a multitude of neuropsychiatric defects, including depression, autism, schizophrenia, Alzheimer’s disease, and epilepsy. Therefore, these receptors represent important therapeutic targets for many CNS disorders, and both negative and positive regulators have potential roles. Oxysterol compounds, analogs of the natural cholesterol metabolite 24S-hydroxycholesterol (24S-HC), are pharmacologically efficacious positive allosteric modulators of NMDARs (Paul et al., 2013; Linsenbardt et al., 2014; Warikoo et al., 2018). 24S-HC is the most abundant cholesterol oxidation product in the brain, reaching concentrations that modulate NMDAR function (Sun et al., 2016a). Synthetic and natural oxysterols are still being characterized, but oxysterol analogs may hold promise in the treatment of neuropsychiatric disease (Sun et al., 2016b; Warikoo et al., 2018). Pharmacological actions of lipophilic agents like oxysterols can be affected by cellular compartmentalization, which may serve as a source or a sink of drug actions, depending on circumstances (Li et al., 2007; Jiang et al., 2016). However, the mechanisms by which oxysterols are trafficked or compartmentalized in neurons are unknown. A limitation to understanding these mechanisms is the lack of methods to visualize the cellular distribution of oxysterols. To reduce this barrier, we introduce a novel analog of 24S-HC, MQ-182. This analog contains a diazirine group, allowing for *in situ* photolabeling, and an alkyne group, permitting click chemistry. The approach of combining click chemistry and photolabeling has recently emerged as a tool for studying cyto-localization (Hofmann et al., 2014; Peyrot et al., 2014; Jao et al., 2015), and our group has previously used this approach to characterize the cellular actions of GABA-active neurosteroid analogs (Jiang et al., 2016). These analogs may also prove useful for identifying sites on NMDARs and other targets for steroid and oxysterol binding (Chen et al., 2012, 2014; Budelier et al., 2017). In this study, we introduce a click photolabeling analog MQ-182 to probe cellular compartmentalization, thereby informing our understanding of oxysterol uptake and accumulation.

Here, we characterize the pharmacological effects, using an *in vitro* electrophysiological approach, and cellular accumulation of MQ-182 in cultured hippocampal neurons. Chemical modification required for visualization may alter the behavior of an analog, so we assayed the properties of MQ-182 at NMDARs and several off-target candidates. We demonstrate that MQ-182 is a potent potentiator of NMDARs, increases the open channel probability of the NMDAR, and occludes the effect of another oxysterol-like modulator but not a sulfated steroid modulator. These observations suggest that the analog is biologically active and modulates NMDARs through an oxysterol-like mechanism. MQ-182 accumulates diffusely in the cytoplasm of neurons, suggesting the possibility of additional targets. At high concentrations, MQ-182 unexpectedly potentiated GABA release, but at lower concentrations, its effects were confined to NMDAR modulation.

Our work introduces a synthetic analog that retains electrophysiological characteristics of oxysterols while enabling visualization of drug localization. The intracellular accumulation of this probe may be relevant to cellular actions of oxysterols aside from an effect on surface NMDARs, although our work revealed only limited evidence for off target effects at high concentrations. Our approach of tandem photolabeling and click chemistry offers the promise of biochemical studies to identify oxysterol sites on NMDARs.

## Materials and Methods

### Hippocampal Cell Culture

Cell cultures were prepared and maintained as described previously (Mennerick et al., 1995). In brief, hippocampal and cortical tissue was harvested from postnatal 1–3 days old Sprague–Dawley rats of both sexes. This study was carried out in accordance with the recommendations of the National Institutes of Health guidelines for animal care and use. The protocol was approved by the Washington University Institutional Animal Care and Use Committee.

Murine neuro-2a (N2a; ATCC #CCL-131) cells were cultured in Dulbecco’s Modified Eagle’s medium (DMEM) with 10% FBS, 2 mM glutamine, 100 U/ml penicillin, and 0.1 mg/ml streptomycin in 5% CO_2_ and 95% air. Cells were transiently transfected with GluN1a (0.34 μg) and GluN2A-GluN2D subunits as previously described (Warikoo et al., 2018).

### Electrophysiology

Whole-cell electrophysiological recordings were performed on the stage of an Eclipse TE2000-S inverted microscope. Data were collected using with a Multiclamp 700B amplifier and Digidata 1440 data acquisition board (Molecular Devices) using pClamp 10 software. During experiments in which GABAergic and glutamatergic postsynaptic currents (PSCs) were studied, the intracellular pipette solution contained the following (in mM): 130 CsCl, 4 NaCl, 10 HEPES, 5 EGTA, and 0.5 CaCl_2_. The pH was adjusted to 7.25 with NaOH. In all other experiments, the solution contained 130 CsMeSO_4_ instead of CsCl.

Extracellular solution during whole-cell patch clamp recordings typically contained 138 mM NaCl, 4 mM KCl, 10 mM HEPES, 10 mM glucose, 2 mM CaCl_2_, 1 mM MgCl_2_, 1 μM NBQX, and 10 μM gabazine. During experiments in which GABAergic PSCs were recorded from neurons, 25 μM D-APV was used instead of 10 μM gabazine, and during experiments in which AMPAR PSCs were recorded, 25 μM D-APV was used instead of 1 μM NBQX. During experiments in which miniature postsynaptic currents (mPSCs) were recorded, 0.25 μM TTX was added. Antagonists were omitted in recordings from transfected N2a cells.

Whole-cell recording pipettes were pulled from borosilicate glass capillary tubes (World Precision Instruments) and had final open-tip resistances of 3–6 MΩ. Neurons were clamped at -70 mV unless otherwise stated. When drug delivery was performed, solutions were dispensed by a gravity-driven local perfusion system from a common tip with exchange time of ∼100 ms.

### *In situ* Click Chemistry and Imaging

Cell cultures were incubated with 10 μM MQ-182 in saline containing (in mM): NaCl (138), KCl (4), CaCl_2_ (2), MgCl_2_ (1), glucose (10), HEPES (10), pH 7.25 with NaOH. Cells were exposed to 365 nm light from a 0.15A Blak-Ray lamp for 15 min. Afterward, cells were fixed for click processing with 4% paraformaldehyde + 0.05% glutaraldehyde in phosphate-buffered saline (PBS) for 10 min. In some experiments, cells were fixed for 10 min prior to incubation in MQ-182 with 15 min ultraviolet (UV) illumination. After rinsing with PBS, the click reaction proceeded by incubating fixed cells with 1 μM azide-Alexa Fluor 488 (Molecular Probes), a click reagent for fluorescence visualization, in 100 μM Tris[(1-benzyl-1*H*-1,2,3-triazol-4-yl)methyl]amine in DMSO, 2 mM sodium ascorbate, and 1 mM CuSO_4_ for 1 h in the dark. For combined *in situ* click and immunostaining, protocols were as described previously (Jiang et al., 2016). Briefly, prior to *in situ* click labeling, cells were incubated in primary antibodies against PDI (1:2000) and giantin (1:2000), for 2 h, followed by labeled secondary antibody (AlexaFluor 647, 1:500) for 1 h.

Cells were imaged on a Nikon Eclipse TE2000-S microscope equipped with a C1 laser scanning confocal attachment. For each field, a z-stack image was acquired, and a maximum intensity z-projection was created from the slices wherein the cells of interest were visible. In order to assess peri-membrane and intracellular fluorescence, region of interest (ROI) lines at 10 pixel thickness were drawn through the cell from left to right, excluding the nucleus. Fluorescence intensity was measured along the line, and fluorescence values were divided into three equal parts. Peak fluorescence value of the first third of pixels above background fluorescence was taken as the intensity of the left cell membrane, average fluorescence of the next third of pixels was taken as the intracellular fluorescence, and peak fluorescence of the final third of pixels was taken as the fluorescence of the right cell membrane. In the case of peak membrane fluorescence, values were validated by eye to occur near the edge of the cell. Quantitation of confocal images was performed using the Fiji distribution of ImageJ imaging software. Fluorescence intensity from individual experiments was log_10_ transformed to better meet the assumption of data normality of parametric tests. Analysis of variance or linear mixed models using cluster (experiment) as a separate covariate were performed to test for fluorescence differences among conditions.

### Data

Data were analyzed and plotted using Clampfit 10 (Molecular Devices), Excel 2011 (Microsoft), Prism 6 (GraphPad), SAS 9.4 (SAS Institute Inc.), and ImageJ software. Unless otherwise stated, summary data in figures and text are given as mean ± SEM. Statistical tests for comparison of means are described in the figure legends for clarity. Comparisons were corrected for multiple comparisons as described in the legends. Statistical significance was defined as a corrected *p-*value < 0.05. The reported *n* refers to the number of neurons in each group within a particular experiment. For imaging experiments, the number of independent experiments is also given.

### Materials

The synthesis of MQ-182 is reported in the Supplementary Materials. D-APV, NBQX, and TTX were obtained from Tocris Biosciences, and all materials without an identified supplier were obtained from Sigma-Aldrich. Antibody species and sources were as follows: giantin (Covance Research Products Inc catalog PRB-114C-200, RRID:AB_10063713), PDI (Abcam catalog ab2792, RRID:AB_303304). SGE-201 was a gift from Sage Therapeutics.

## Results

### MQ-182 Is a Positive Allosteric Modulator at NMDARs

**FIGURE 1:**
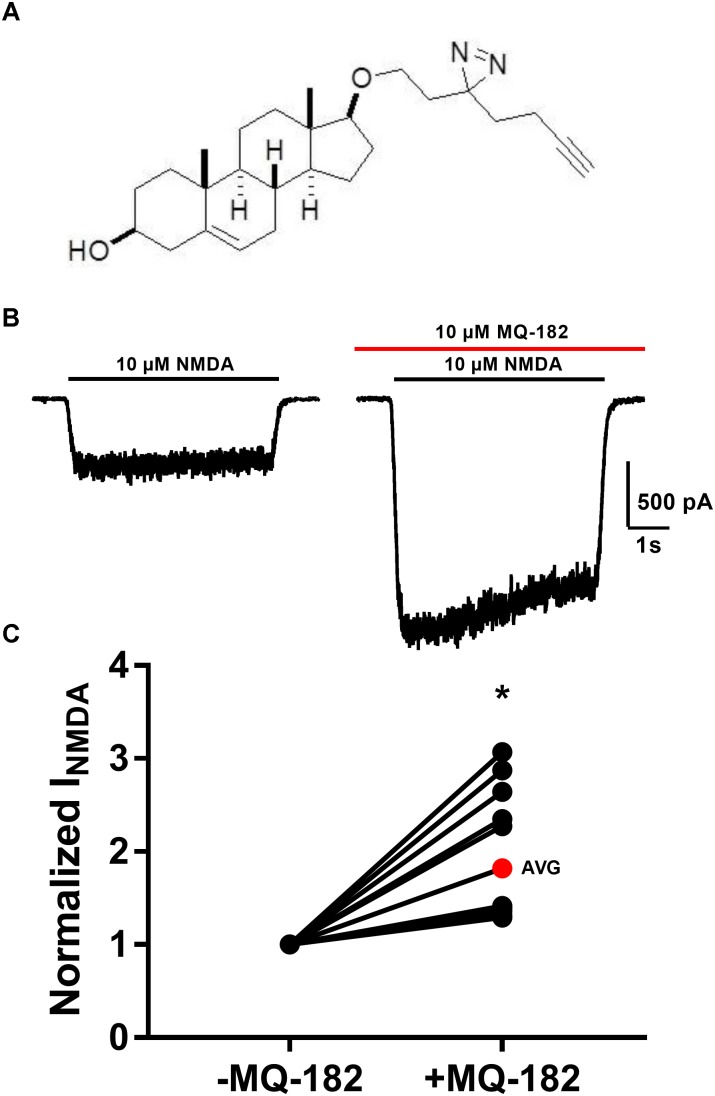
MQ-182 is a potent positive allosteric modulator of NMDARs. **(A)** Natta projection structure of MQ-182. **(B)** Effect of MQ-182 on NMDAR currents. Cultured primary hippocampal neurons were incubated in 10 μM MQ-182 and 20 μM D-serine in the nominal absence of Mg^2+^ for 30 s, followed by 5 s NMDA application. The horizontal black bar represents the application of NMDA (10 μM, 5 s) and the red bar represents the application of MQ-182 (10 μM, 45 s). Pre-application was for 30 s before co-application with NMDA. **(C)** The normalized change in NMDAR current magnitude is plotted. The red symbol denotes the mean potentiation. Asterisk represents a significant increase in NMDAR current magnitude (*p* = 0.00020, *n* = 15, one-sample *t*-test).

**FIGURE 2:**
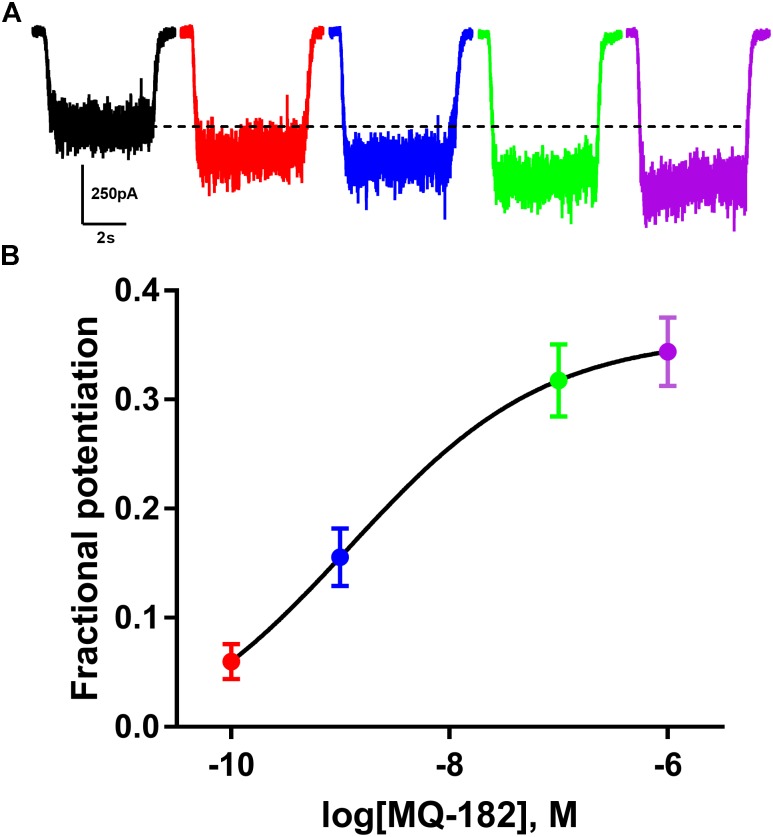
MQ-182 is effective at sub-micromolar concentrations. **(A)** Increasing potentiation of NMDAR currents (10 μM NMDA, 20 μM D-serine) by increasing concentrations of MQ-182. Concentrations used were 0.1 nM (red), 1 nM (blue), 0.1 μM (green), 1 μM (purple), pre-applied before agonist application. Dotted line represents magnitude of baseline NMDA current. **(B)** Potentiation values were fit with the Hill equation (solid line). EC_50_ of MQ-182 was estimated at 1.2 nM (*n* = 14). Symbols are color coded as in **(A)**.

The structure of MQ-182 is shown in Figure 1A. In the sterol side chain, the compound contains a diazirine group, enabling photoattachment to lipids and proteins in the presence of 365 nm UV light, and an alkyne group, enabling bio-orthogonal copper catalyzed azide-alkyne Huisgen cycloaddition. Chemical modifications can alter the physiological behavior of a compound, so we assayed MQ-182 at NMDARs to determine if it retained oxysterol-like activity. In dissociated cultures of rat hippocampal neurons, we explored the effect of MQ-182 on currents evoked by exogenous NMDA (Figures 1B,C). MQ-182 (10 μM) pre-application caused nearly a doubling of the response to 10 μM NMDA, consistent with the actions of naturally occurring oxysterols (Paul et al., 2013). In a concentration-response study, MQ-182 potentiated NMDAR currents at low nanomolar concentrations, with an EC_50_ estimate of 1.2 nM (Figure 2). This is ∼600- and 60-fold more potent than 24S-HC and SGE-201, a natural and synthetic oxysterol analog, respectively (Paul et al., 2013).

**FIGURE 3:**
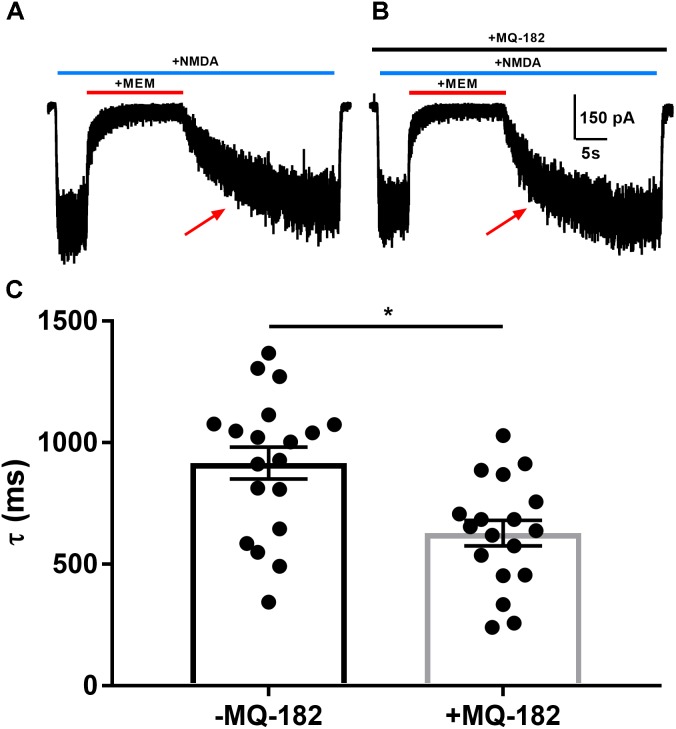
MQ-182 accelerates rate of memantine block reversal, suggesting that it increases the open-channel probability of the NMDAR. **(A)** Cells were challenged with 10 μM NMDA (horizontal blue bar, 40 s) and 10 μM memantine (MEM, horizontal red bar, 15 s). **(B)** Cells were pre-incubated in 10 μM MQ-182 for >5 min and challenged with the same protocol as described in **(A)**. **(C)** The current relaxation upon memantine removal (red arrows in **A**) was fitted with a monoexponential curve. Time constant values (τ) from these fits are plotted. Asterisk represents a decrease in tau (*p* = 0.0017, *n* = 19 for -MQ-182, *n* = 18 for +MQ-182, Student’s unpaired *t*-test).

Oxysterols increase NMDAR channel open probability, resulting in altered kinetics of dissociation of open-channel trapping blockers such as memantine and ketamine (Emnett et al., 2015), which require channel opening for dissociation. To characterize further MQ-182’s potentiation of NMDAR currents, we explored its effects on the rate of open-channel blocker dissociation (Figure 3). After obtaining a steady-state NMDAR current, we co-applied memantine, which rapidly decreased NMDAR current. When the current reached a new steady state, we removed memantine in the continued presence of NMDA and evaluated the kinetics of re-emergence of current to its original magnitude. In the presence of MQ-182, the time constant of current re-emergence was decreased relative to different cells in the absence of MQ-182 (Figure 3C). This change suggests that MQ-182 increased the open-channel probability of the NMDAR, thereby accelerating the reversal of memantine’s actions.

**FIGURE 4:**
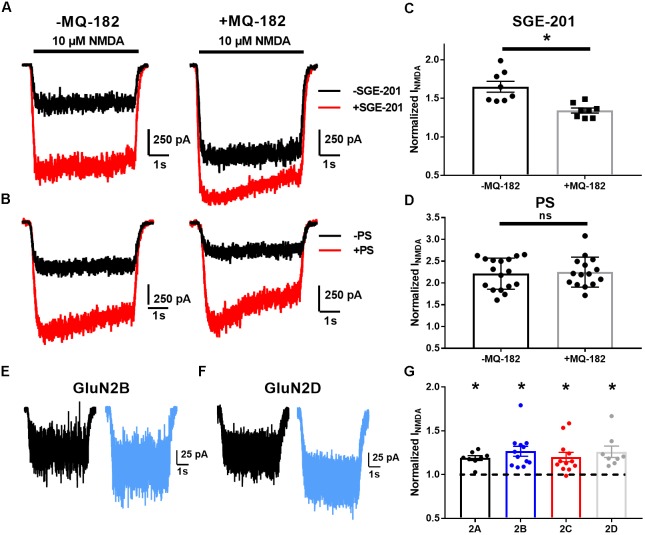
MQ-182 exhibits characteristics of oxysterol potentiation rather than sulfated steroid potentiation. **(A–D)** Occlusion studies. **(A,B)** Representative traces of occlusion protocol. Cells were preincubated in MQ-182 for >5 min and then challenged with 10 μM NMDA (5 s, horizontal black bar) and 0.2 μM SGE-201 or 50 μM PS (45 s pre-application). Each pair of black and red traces comes from a different cell. **(C,D)** Summary data of **(A,B)**. Although SGE-201’s potentiation of NMDARs was decreased by pre-incubation in MQ-182, PS’s potentiation was not (*p* = 0.78, *n* = 17 for -MQ-182, *n* = 15 for +MQ-182). Asterisk represents *p* = 0.0013, *n* = 8 (potentiated response vs. baseline), Student’s unpaired *t-*test. **(E–G)** Tests of subunit selectivity. **(E,F)** N2A cells expressing human GluN1a and one of four GluN2 subunits **(A–D)** were incubated in 10 μM MQ-182 and 20 μM D-serine in the nominal absence of Mg^2+^ for 30 s, followed by 5 s NMDA application. Representative traces are shown for two cells in **(E,F)**. **(G)** Subunits containing each of the four GluN2 subunits (GluN2A-D) showed potentiation by MQ-182. Dotted line represents magnitude of baseline NMDA current. Asterisks represent a significant increase in NMDAR current magnitude (asterisks: GluN2A, *p* = 0.00020, *n* = 8; GluN2B, *p* = 0.00070, *n* = 12; GluN2C, *p* = 0.0032, *n* = 12; GluN2D, *p* = 0.0053, *n* = 8; one-sample paired *t*-test).

Increased probability of channel opening characterizes several classes of positive NMDAR modulators (Popescu, 2005). The actions of oxysterol-like modulators can be distinguished from others by occlusion assays (Linsenbardt et al., 2014). We tested pharmacological occlusion by pre-incubating neurons for >5 min in 10 μM MQ-182 and then challenging with NMDA and another positive allosteric modulator (Figure 4). SGE-201, a synthetic oxysterol analog, is a potent potentiator of the NMDAR, and we would expect a decrease of its potentiation if MQ-182 interacts with the oxysterol binding site. Indeed, MQ-182 incubation decreased SGE-201 potentiation, but not pregnenolone sulfate (PS) potentiation (Figures 4C,D). We note that the occlusion between SGE-201 and MQ-182 was incomplete at the concentrations tested (Figure 4). This could suggest additional mechanisms, but the data suggest at least a partially shared mechanism of action between MQ-182 and SGE-201, supporting an oxysterol-like mechanism.

Another distinguishing characteristic of oxysterol-like potentiation vs. PS-like potentiation is subunit selectivity. Oxysterols potentiate GluN2C and GluN2D-containing NMDARs (Paul et al., 2013), but PS does not (Horak et al., 2006). Consistent with the idea that MQ-182 is an oxysterol-like modulator, we found that 10 μM MQ-182 potentiated recombinant receptors containing GluN2A, B, C, or D (Figures 4E–G).

**FIGURE 5:**
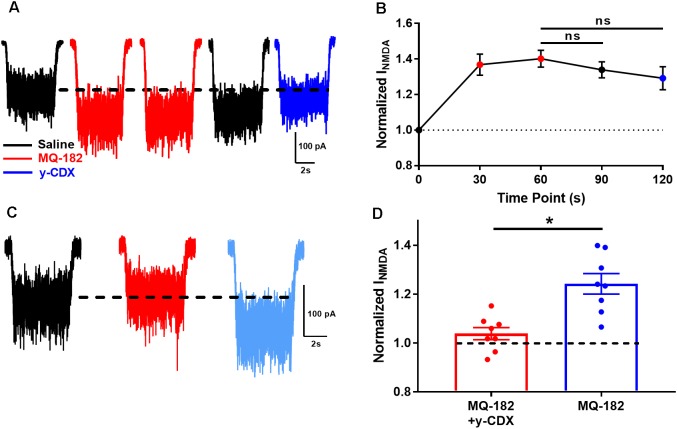
The actions of MQ-182 exhibit weak reversibility. **(A)** Cells were challenged with 10 μM NMDA to establish baseline NMDAR currents. Cells were then challenged with repeated 30 s pre-applications of 0.5 μM MQ-182, a sub-saturating concentration (red traces). Following 60 s total MQ-182 exposure, cells were challenged with 30 s saline wash (black trace). Cells were then challenged with γ-CDX wash (30 s, 500 μM) before application of NMDA in the absence of γ-CDX. **(B)** Summary plot. The Friedman test, a modified one-way repeated measures ANOVA for non-parametric data, revealed no interaction between the treatment condition (MQ-182, saline wash, γ-CDX wash) and magnitude of NMDAR current (*p* = 0.37, *n* = 5). *Post hoc* Dunn’s multiple comparisons tests revealed no differences between the MQ-182 and saline wash conditions (*p* = 0.42, *n* = 5), or between the MQ-182 and γ-CDX wash conditions (*p* = 0.23, *n* = 5). **(C)** Test of CDX/MQ-182 interaction. Cells were first challenged with 10 μM NMDA (black trace), and baseline NMDAR currents were established. Next, they were challenged with a solution of 0.5 μM MQ-182, pre-mixed with 500 μM γ-CDX (red trace). Finally, they were challenged with 0.5 μM MQ-182 without γ-CDX (light blue trace). **(D)** Comparison of potentiation by MQ-182 alone and MQ-182 pre-mixed with γ-CDX. Dotted line represents magnitude of baseline NMDA current. A Wilcoxon matched-pairs signed rank test showed a difference between conditions. Asterisk represents an increase in potentiation from the MQ-182 + γ-CDX condition to the MQ-182 alone condition (*p* = 0.0078, *n* = 8). Dashed line represents baseline NMDA current.

We observed in pilot experiments that potentiation took ∼30 s to reach maximal effect, and was poorly reversible once established. It is possible that MQ-182’s slow kinetics are due to slow dissociation from the NMDAR, or from slow departitioning from the plasma membrane bilayer, from which it likely accesses the NMDAR. If the membrane-associated compound is in rapid equilibrium with the receptor, extracellularly applied γ-cyclodextrin (γ-CDX) may be able to complex and sequester MQ-182 (Shu et al., 2007), leading to accelerated offset of MQ-182 effects. To investigate this, we potentiated NMDAR currents with two 30 s pre-applications of 0.5 μM MQ-182, followed by co-application with NMDA (Figure 5A). We used 0.5 μM MQ-182 instead of 10 μM to avoid saturation. After assuring maximum potentiation, cells were washed with 30 s saline before re-application of NMDA alone. Saline wash had little effect on the potentiated NMDAR current, confirming slow reversibility (Figure 5B). Cells were then challenged with γ-CDX (500 μM), followed by re-application of NMDA. γ-CDX application did not reduce potentiated NMDAR current. This suggests one of at least three possibilities: (1) that MQ-182 rapidly dissociates from the NMDAR but resides in the membrane at a depth inaccessible to γ-CDX, (2) that MQ-182 binds to the receptor with a slow dissociation rate, or (3) that MQ-182 does not easily complex with γ-CDX. This latter possibility has been shown for the natural oxysterol 24S-HC (Paul et al., 2013).

To experimentally test the latter possibility, we pre-mixed MQ-182 with γ-CDX (Figure 5C). If MQ-182 does not form inclusion complexes with γ-CDX, then we would expect MQ-182 pre-mixed with γ-CDX to exhibit reduced potentiation compared with MQ-182 alone, since γ-CDX-complexed MQ-182 would presumably not be active. Indeed, we observed that MQ-182 potentiation was reduced compared to a control MQ-182 solution containing no γ-CDX (Figure 5D). These results suggest that MQ-182 binds avidly to the NMDAR or partitions into the membrane at a position inaccessible to γ-CDX. These alternatives are considered further in the Discussion.

### MQ-182 Intracellular Accumulation

**FIGURE 6:**
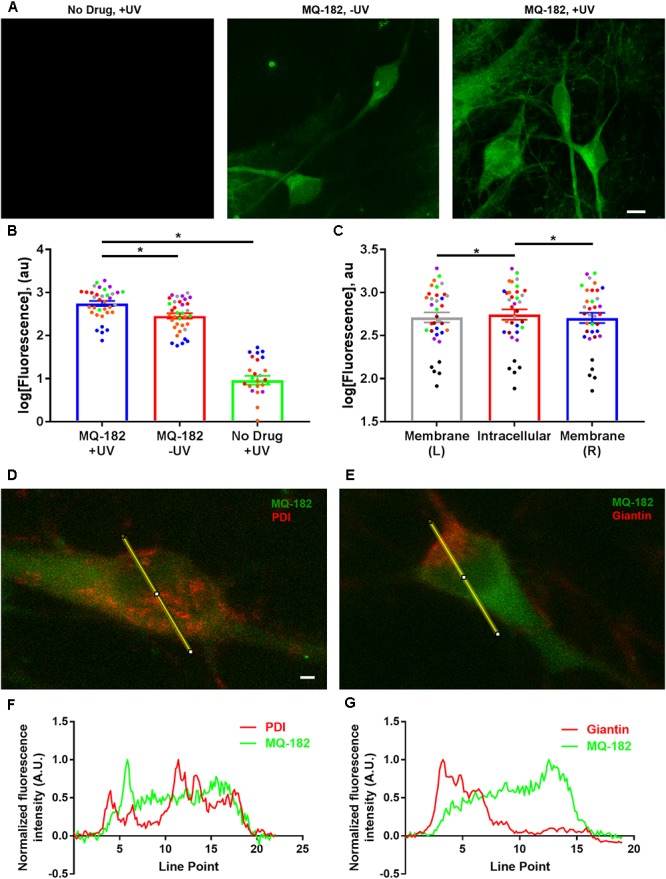
Diffuse cytoplasmic labeling of a hippocampal neuron by MQ-182. **(A)** A hippocampal culture was incubated in 10 μM MQ-182, exposed to 365 nM UV irradiation for 15 min, and processed for click cyto-fluorescence using azide-conjugated AlexaFluor 488. Panels are labeled with the experimental conditions. Scale bar: 15 μm. **(B)** Comparison of MQ-182 +UV, MQ-182 -UV, and no drug +UV conditions. A two-way ANOVA with experimental condition and day of experiment (represented as separate colored symbols) as the two independent variables revealed a significant difference in fluorescence between experimental conditions [*p* < 1.0 × 10^-15^, *n* = 35, *F*_(2,75)_ = 523.3]. There was also a significant difference between experiment [*p* = 6.2 × 10^-12^, *n* = 35, *F*_(6,75)_ = 16.34] and an interaction between experimental condition and experiment [*p* < 1.0 × 10^-15^, *n* = 35, *F*_(12,75)_ = 16.44], suggesting differences in the degree of overall fluorescence in different experimental runs. *Post hoc* Bonferroni corrected Student’s unpaired *t-*tests revealed significant differences between the MQ-182 +UV and MQ-182 -UV conditions [*p* = 1.1 × 10^-7^, *n* = 35, bottom asterisk) and between the MQ-182 +UV and the no drug +UV conditions (*p* < 1.0 × 10^-15^, *n* = 35, top asterisk). **(C)** Comparison of peri-membrane and intracellular fluorescence in the MQ-182 +UV condition. Membrane (L) refers to the left side of the cell membrane as viewed in the z-projection, while Membrane (R) refers to the right side. A two-way ANOVA suggested differences between intracellular and peri-membrane fluorescence [*p* = 0.0048, *n* = 35, *F*_(2,__56)_ = 5.88]. *Post hoc* Bonferroni corrected Student’s unpaired *t-*tests revealed differences between the intracellular and left membrane locations (*p* = 0.019, *n* = 35, left asterisk) and between the intracellular and right membrane locations (*p* = 0.0046, *n* = 35, right asterisk), suggesting somewhat stronger intracellular fluorescence. **(D,E)** Combined click cyto-fluorescence with immunofluorescence for PDI, an endoplasmic reticulum marker, and giantin, a Golgi-specific protein, show little co-labeling. Scale bar: 2.14 μm. Line scans are shown in (**F,G).**

After investigating the pharmacological behavior of MQ-182, we performed *in situ* visualization of the analog using tandem photolabeling and click chemistry (Figure 6). Cells were incubated in 10 μM MQ-182 with or without UV light exposure, followed by paraformaldehyde fixation. Because diazirine photolabeling covalently attaches compounds to nearby proteins and lipids, this protocol permanently labels intracellular sites of steroid accumulation with untagged MQ-182. Following fixation and membrane permeabilization, we used azide-conjugated Alexa Fluor 488 for click-mediated fluorescence visualization. Figure 6B shows that both UV light and the presence of MQ-182 are required for strong labeling of hippocampal neurons, confirming the effectiveness of the *in situ* click reaction and the *in situ* photolabeling. We also observed labeling without UV illumination, indicating strong intracellular retention even in the absence of photolabeling (Figures 6A,B). The pattern of accumulation was mainly somatic with little neuritic labeling, consistent with previous studies using fluorescent neurosteroid analogs (Jiang et al., 2016). MQ-182 accumulated diffusely in the cytoplasm of neurons, with minor differences between intracellular and peri-membrane fluorescence (Figure 6C). The lack of plasma-membrane enrichment was also evident in co-labeling experiments in which Golgi was labeled with anti-giantin antibody, and endoplasmic reticulum was labeled with anti-PDI antibody (Figure 6D). Neither antibody showed significant co-localization (Figures 6E,F), suggesting indiscriminate intracellular accumulation by MQ-182. This pattern of accumulation differs from that of other neurosteroid analogs, with many exhibiting Golgi-selective or membrane-selective accumulation (Peyrot et al., 2014; Jiang et al., 2016).

**FIGURE 7:**
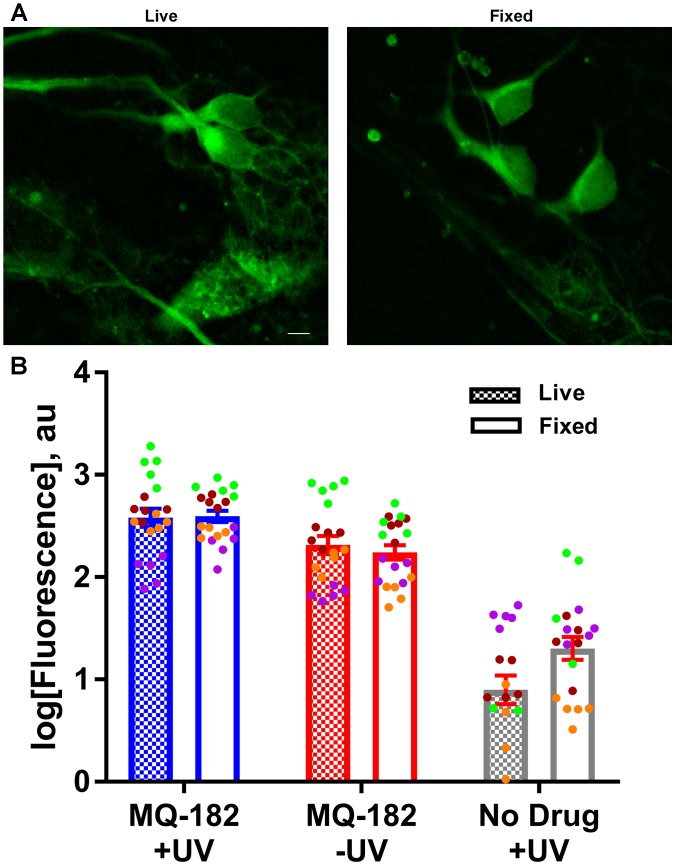
MQ-182 accumulation in fixed neurons. **(A)** Neurons were incubated with 10 μM MQ-182 and exposed to the UV irradiation protocol while either alive or fixed in 4% paraformaldehyde. Live cells were then fixed in paraformaldehyde, and all cells were processed for click cyto-fluorescence as previously described. Panels depict live and fixed cells in the MQ-182 +UV condition. Scale bar: 5 μm. **(B)** Summary of labeling intensity. A linear mixed model revealed no difference in fluorescence between the live and fixed conditions when adjusting for day of experiment and presence of drug/UV [*p* = 0.1192, *n* = 20, *F*_(1,110)_ = 2.47]. Each independent experiment is denoted with a different color symbol.

To gain insight into factors that might contribute to MQ-182’s diffuse pattern of accumulation and retention, we investigated whether the uptake of MQ-182 was energy dependent (Figure 7). In this experiment, we compared fixed neurons, which cannot use ATP-dependent pathways, with live neurons. Interestingly, there was little change in either the intensity of labeling or the pattern of accumulation (Figure 7B). We conclude that energy-dependent pathways do not need to be invoked in the intracellular accumulation of MQ-182.

**FIGURE 8:**
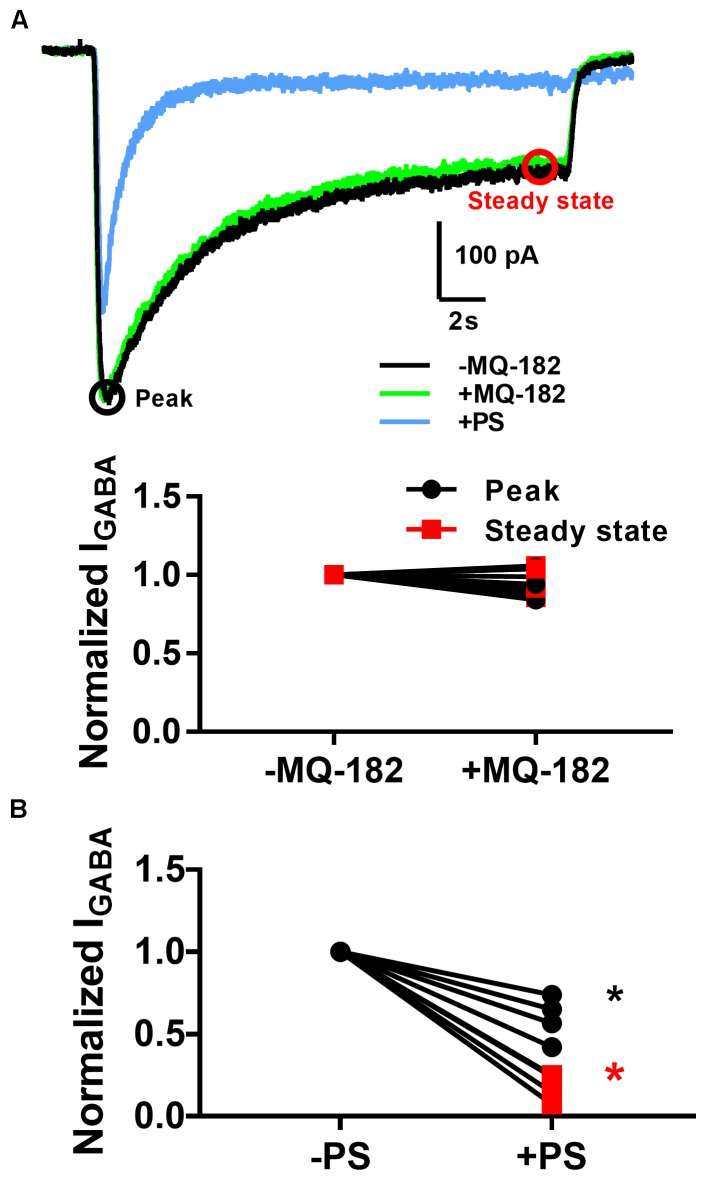
MQ-182 does not potentiate responses to exogenous GABA. **(A)** Hippocampal cultures were incubated in 10 μM MQ-182, 25 μM D-APV and 1 μM NBQX for 30 s, followed by 20 s application of 5 μM GABA (black). Baseline GABA_A_R currents were established. Cells were then challenged with 30 s pre-incubation of MQ-182, followed by 20 s application of GABA co-applied with MQ-182 (green). Finally, they were challenged with 20 s application of GABA co-applied with 10 μM PS (blue). Representative traces are shown, with peak and steady state areas of the trace circled with colors used for ensuing summaries. **(B)** Summary of effect of drugs on peak and steady-state GABA current. A two-way repeated measures ANOVA indicated a main effect of drug for PS (*p* = 0.009) but not for MQ-182 (*p* = 0.70). For PS there was also a significant interaction between drug and time (*p* = 0.009), indicating a more prominent effect on steady-state current, as expected from the mechanism of PS modulation of NMDARs (Eisenman et al., 2003).

The diffuse pattern of accumulation also led us to wonder whether MQ-182 might have neuronal targets besides NMDARs. In order to investigate possible off-target effects, we recorded pooled spontaneous AMPA receptor (AMPAR) spontaneous EPSCs (sEPSCs) and GABA_A_ receptor (GABA_A_R) sIPSCs from multineuron cultures. Because PSCs often occur in bursts, which precluded accurate measurement of event frequency, we integrated the total negative-going current as a measure of aggregated presynaptic activity and postsynaptic effects. We observed in pilot experiments that at 10 μM, MQ-182 increased the total charge of sPSCs. Because some steroid-like neuromodulators interact with GABA_A_Rs, we hypothesized that MQ-182 increased the frequency of sPSCs by blocking network inhibition. However, the effect did not result from postsynaptic disinhibition because 10 μM MQ-182, in contrast to PS, failed to affect exogenous GABA responses (Figure 8).

**FIGURE 9:**
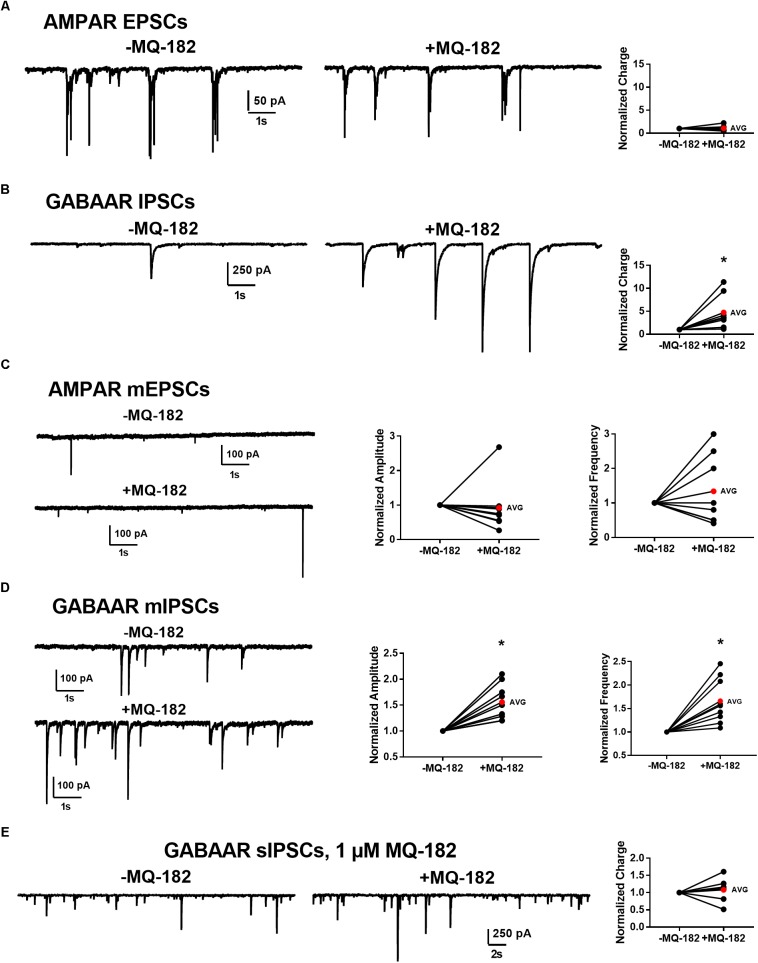
Effect of MQ-182 on sPSCs and mPSCs. **(A,B)** Effect on AMPAR ESPCs **(A)** and GABA_A_R IPSCs **(B)**. **(A)** In 10 μM gabazine and 25 μM D-APV to isolate AMPAR EPSCs, baseline sEPSCs were followed by 30 s pre-application of 10 μM MQ-182 and further recording. Because many EPSCs occurred in burst-like clusters in which individual events could not be accurately discerned, currents were integrated to yield total negative-going charge. The total charge of AMPAR EPSCs did not increase. The right plot shows effect of MQ-182 normalized to baseline for individual cells. The red symbol denotes the mean change. ( **(B)** Hippocampal cultures were exposed to 1 μM NBQX and 25 μM D-APV to isolate GABA_A_R IPSCs and the MQ-182 application protocol was repeated. The total charge of GABA_A_R IPSCs was increased. Asterisk represents a significant increase in total charge (*p* = 0.024, *n* = 8, one-sample *t*-test). **(C,D)** Selective potentiation of GABA_A_R mIPSCs. AMPAR mEPSCs **(C)** were isolated with 10 μM gabazine, 25 μM D-APV, and 0.2 μM TTX. Neither the amplitude nor the frequency of the mEPSCs were potentiated by MQ-182. GABA_A_R mIPSCs **(D)** were isolated with 1 μM NBQX, 25 μM D-APV, and 0.2 μM TTX. Both the amplitude and the frequency of GABA_A_R mIPSCs were potentiated by 10 μM MQ-182. Left asterisk represents a significant increase in amplitude (*p* = 0.0011, *n* = 9, one-sample *t*-test). Right asterisk represents a significant increase in frequency (*p* = 0.0034, *n* = 9, one-sample *t*-test). **(E)** Integrated total charge of GABA_A_R IPSCs was not potentiated by 1 μM MQ-182. GABA_A_R IPSCs were isolated as in **(B)**. Recording in the presence of 1 μM MQ-182 followed a 30 s application. Application of 1 μM MQ-182 did not increase the total negative-going charge of GABA_A_R IPSCs (*p* = 0.49, *n* = 8, one-sample *t*-test).)

To determine whether effects on PSCs occur at both excitatory and inhibitory synapses, we pharmacologically isolated AMPAR transmission and GABA_A_R transmission individually and challenged cells with 10 μM MQ-182. We found that while GABA_A_R sIPSC charge was potentiated, AMPAR sEPSC charge was not (Figures 9A,B).

These effects could be mediated by increased interneuron firing or by direct effects on synaptic function. To test whether the effect of 10 μM MQ-182 on spontaneous transmission arose from direct action on synaptic targets, we examined mPSCs. MQ-182 (10 μM) increased both frequency and amplitude of GABA_A_R mIPSCs but did not affect AMPAR mEPSCs (Figures 9C,D). These results suggest complex actions of 10 μM MQ-182 involving presynaptic targets at inhibitory synapses.

**FIGURE 10:**
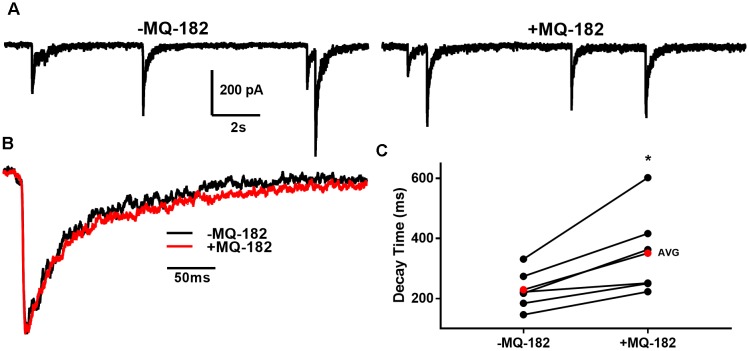
MQ-182 potentiates NMDAR sEPSCS. **(A)** In 10 μM gabazine and 1 μM NBQX to isolate NMDARs, baseline sEPSCs were followed by 30 s pre-application of 1 μM MQ-182 and further recording. Because the majority of NMDAR sEPSCs did not occur in burst-like clusters, like GABA_A_R sIPSCs, individual EPSCs could be detected with confidence. **(B)** Representative trace of an average waveform of individual EPSCs for an exemplar cell. **(C)** EPSC decays were fitted with a bi-exponential function. Tau values weighted by the relative amplitudes of the components are plotted. The red symbols denote the mean decay time for each condition. MQ-182 increased the decay time of NMDAR ESPCs. Asterisk represents potentiation (*p* = 0.018, *n* = 6, Student’s paired *t-*test). Amplitude of events was not increased (-1.96 ± 5.92% increase, *p* = 0.71, *n* = 6, Student’s paired *t-*test). Frequency of events was also not increased (-2.65 ± 7.32% increase, *p* = 0.49, *n* = 6, Student’s paired *t*-test).

To determine if these off-target effects are preserved at lower concentrations of MQ-182 at which NMDAR modulation is observed, we tested 1 μM MQ-182 on sIPSCs. We did not observe a potentiation in charge (Figure 9E), suggesting that MQ-182’s actions at GABA synapses are not observed at concentrations still near maximum for NMDAR effects. Although 1 μM MQ-182 did not effect a reliable potentiation of GABA_A_R sIPSC charge, it increased the decay time constant of NMDAR sEPSCs (Figure 10), confirming selective modulation of NMDARS at this concentration.

## Discussion

Oxysterol analogs are candidates for the treatment of neuropsychiatric disorders (Sun et al., 2016b; Warikoo et al., 2018). To successfully develop these compounds as therapeutics, we must find tools to explore their cellular fate. However, the methods available for the microscopic imaging of sterols are few (Jao et al., 2015). Here, we studied the pharmacological properties and cellular accumulation of a visualizable oxysterol analog. This analog retains physiological activity at NMDARs and permits tandem photolabeling and click chemistry, allowing us to permanently label sites of accumulation, followed by visualization. The studies demonstrate potent, long-lived actions of MQ-182 and ample intracellular accumulation without discernible plasma membrane enrichment.

To validate MQ-182 as an oxysterol analog, we assayed physiological properties at NMDARs. We found that MQ-182 behaves, similarly, to 24S-HC, a major brain cholesterol metabolite, at NMDARs. Both MQ-182 and 24S-HC potently potentiate NMDAR currents, increase the channel open probability of the NMDAR, and exhibit slow reversibility. Furthermore, MQ-182 reduces potentiation by SGE-201, another synthetic oxysterol analog, but not PS, a sulfated steroid. Additional neurosteroid analogs may allow additional insight into the structure–activity relationships of allosteric modulators.

The cellular fate of lipophilic neurosteroids and oxysterols is not well understood, with different mechanisms proposed for different oxysterols. For example, some oxysterols accumulate by energy-dependent mechanisms (Peyrot et al., 2014), while others accumulate passively (Jiang et al., 2016). The distribution of steroid-like modulators is also varied – some exhibit Golgi-selective accumulation (Jiang et al., 2016), while others accumulate in other intracellular compartments (Li et al., 2007) or the plasma membrane (Laverty et al., 2017; Miller et al., 2017). Our results suggest a passive mechanism of MQ-182 accumulation, because cell viability was not required for uptake. Co-labeling also suggested a rather indiscriminate intracellular distribution, with failure of co-localization with either Golgi or endoplasmic reticulum markers (Figures 6E,F). This may indicate that lower aqueous concentrations are needed for potentiation, as passive, hydrophobic interactions may promote a high local membrane concentration but could also promote sequestration in intracellular organelles (Chisari et al., 2010). Passive uptake also implies non-specific accumulation in all cells, regardless of expression of transporters or pumps required for active uptake.

Slow reversibility seems to be a hallmark of oxysterol and steroid modulation of NMDAR activity (Horak et al., 2004; Paul et al., 2013). Slow reversibility is also a feature of neuroactive steroids that modulate GABA_A_Rs (Shu et al., 2004). This feature correlates with lipophilicity, consistent with membrane partitioning. In the case of GABAergic neurosteroids, γ-CDX promotes rapid offset of potentiation, suggesting that membrane-associated steroid rapidly dissociates from the receptor into the membrane, where it is accessible to γ-CDX. In the case of MQ-182, we found that potentiation offset is insensitive to γ-CDX. This suggests one of three possibilities: (1) that MQ-182 accumulates in a membrane area inaccessible to γ-CDX, (2) that MQ-182 exhibits slow dissociation from the NMDAR, or 3) that MQ-182 is not sequestered by γ-CDX. Because γ-CDX diminishes potentiation when it is pre-mixed with MQ-182 (Figure 5D), γ-CDX effectively lowers the aqueous concentration of MQ-182, presumably through complexing. Thus, we can exclude the third possibility. We cannot totally exclude possibility 2. However, given that several other lipophilic modulators have recently been shown to have membrane-facing binding sites (Chen et al., 2012; Laverty et al., 2017), and other oxysterols exhibit γ-CDX-sensitive potentiation (Shu et al., 2007), we favor possibility 1, that the interaction of MQ-182 with the membrane precludes access by γ-CDX. MQ-182’s diffuse pattern of accumulation led us to explore off-target effects, and we recorded pooled sEPSCs and sIPSCs from multi-neuron cultures. We observed that 10 μM MQ-182, a saturating concentration at NMDARs, increased the frequency of sPSCs. Because sPSCs are often driven by action potential firing, we reasoned that disinhibition through a postsynaptic effect on GABA_A_Rs might account for the sPSC frequency increase. However, 10 μM MQ-182 failed to inhibit responses to exogenous GABA, indicating that the increase in sPSCs cannot be accounted for by postsynaptic disinhibition. Instead, we found that mIPSC frequency was also increased. Because action potentials are not involved in mIPSCs, we conclude that an effect on presynaptic machinery accounts for the effect of high MQ-182 concentrations on transmission. Our observation that GABA_A_R mIPSCs increase in both amplitude and frequency is unexpected, as an increase in amplitude is classically interpreted as a postsynaptic effect, but MQ-182 fails to alter exogenous GABA_A_R responses (Figure 8). To reconcile these observations, we speculate that MQ-182 may foster multivesicular fusion, leading to elevated synaptic GABA concentration and larger mIPSCs. Although these results merit follow-up, it is important to note that 10 μM MQ-182 is well above the EC_50_ of the compound at NMDARs (1.2 nM). When we tested 1 μM MQ-182, we did not observe an increase in the charge of GABA_A_R sIPSCs, suggesting that MQ-182’s off-target effects are not observed at concentrations closer to the EC_50_ for NMDAR effects. A lower concentration of MQ-182 increased the decay time constant of NMDAR sEPSCs without altering sIPSCs. These results suggest that using MQ-182 at a low dose *in vivo* may selectively modulate NMDARs. However, we anticipate the major uses of MQ-182 will be *in vitro*, for cell biological and biochemical studies in which concentration can be precisely controlled.

A feature of MQ-182 labeling is the retention of some non-UV linked compound following fixation and *in situ* click labeling (Figure 6). This behavior has also been observed for other alkyne-labeled probes (Yang et al., 2010; Viertler et al., 2012; Peyrot et al., 2014; Emnett et al., 2016). The factors resulting in this retention are not clear at present, but qualitatively the distribution of photo-linked compound appeared similar to that of non-photo-linked compound.

In this study, we introduce a novel oxysterol analog that allows tandem photolabeling and click chemistry. This strategy enabled us to permanently label cellular analog accumulation, permitting insight into mechanisms of cellular compartmentalization. Further understanding of this class of compounds should aid in the synthesis of new drugs and to new insights into the targets of biologically active cholesterol metabolites.

## Author Contributions

DMC and SM conceptualized this study. DMC, LZ, and AB performed experiments and obtained data. DFC and MQ synthesized MQ-182 and devised its structure. DMC wrote the original draft of the manuscript. SM, CFZ, and DFC acquired funding for and supervised this study. All authors participated in manuscript revision and approved the submitted version.

## Conflict of Interest Statement

CFZ is a member of the Scientific Board of Sage Therapeutics. DMC and CFZ hold equity in Sage Therapeutics. The remaining authors declare that the research was conducted in the absence of any commercial or financial relationships that could be construed as a potential conflict of interest.
