# Supplementary material for: A Clickable Oxysterol Photolabel Retains NMDA Receptor Activity and Accumulates in Neurons
Source: Front Neurosci. 2018 Dec 6;12:923. doi: 10.3389/fnins.2018.00923 (PMC6291516; doi:10.3389/fnins.2018.00923)
Supplement: Supplementary file 1 [file Data_Sheet_1.PDF]

Supplementary Material

A Clickable Oxysterol Photolabel Retains NMDA Receptor Activity and Accumulates in Neurons

Daniel M. Chen, Luke Ziolkowski, Ann Benz, Mingxing Qian, Charles F. Zorumski, Douglas F. Covey, Steven Mennerick*

*** Correspondence:** Steven Mennerick: menneris@wustl.edu

1 Supplementary Data

2 Supplementary Figures and Tables

2.1 Supplementary Figures

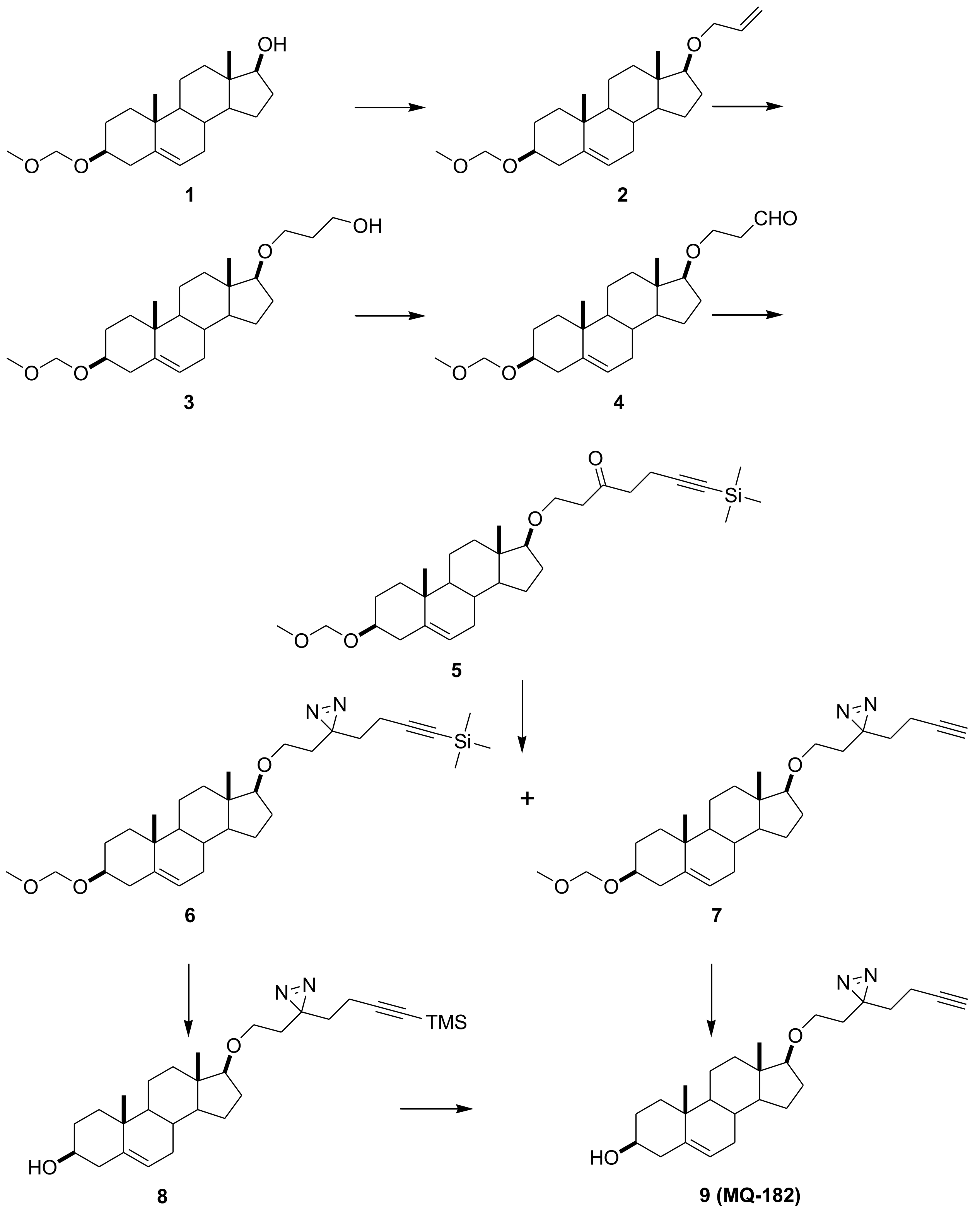

Supplementary Figure 1. Synthetic scheme for MQ-182. **Steroid 2.** To a solution of known steroid **1** ([Vasickova, S.; Pouzar, V.; Cerny, I.; Drasar, P.; Havel, M. *Coll. Czech. Chem. Comm.* **1986**, *51*, 90-100], 2.2 g, 6.59 mmol) in DMF (50 mL) was added NaH (1.2 g, 60% in mineral oil, 30 mmol) and allyl iodide (6.72 g, 40 mmol) at room temperature. After 2 h, water (30 mL) was added and the

mixture was extracted into EtOAc (150 mL x 2). The organic layers were dried, filtered and concentrated. The residue was purified by flash column chromatography (10% of EtOAc in hexanes) to give steroid **2** (2.02 g, 82%): ^{1}H NMR ($CDCl_3$) δ 5.87-5.81 (m, 1H), 5.30 (d, *J* = 4.3 Hz, 1H), 5.22 (d, *J* = 17.2 Hz, 1H), 5.08 (d, *J* = 10.2 Hz, 1H), 4.62 (s, 2H), 3.95 (d, *J* = 5.1 Hz, 2H), 3.39-3.29 (m, 2H), 3.31 (s, 3H), 2.32-0.84 (m, 19H), 0.97 (s, 3H), 0.75 (s, 3H); ^{13}C NMR ($CDCl_3$) δ 140.7, 135.6, 121.3, 115.8, 94.6, 88.3, 70.7, 55.0, 51.5, 50.3, 42.7, 39.5, 37.7, 37.2, 36.7, 31.7, 31.4, 28.8, 27.9, 23.4, 20.7, 19.3, 11.5.

Steroid 3. To a solution of steroid **2** (2.02 g, 5.4 mmol) in THF (60 mL) was added 9-BBN (25 mL, 0.5 M, 12.5 mmol) at room temperature. After 16 h, 3 N NaOH (30 mL) and H_2O_2 (15 mL) were added and stirred for 1 h at room temperature. The product was extracted into EtOAc (100 mL x 2) and washed with brine (50 mL x 2). The combined extracts were dried, filtered and the solvents removed. The residue was purified by flash column chromatography (silica gel eluted with 25% EtOAc in hexanes) to give steroid **3** (1.82 g, 90%): ^{1}H NMR (400 MHz, $CDCl_3$) δ 5.24 (d, *J* = 4.3 Hz, 1H), 4.57 (s, 2H), 3.65-3.60 (m, 2H), 3.54 (t, *J* = 5.9 Hz, 2H), 3.35-3.19 (m, 2H), 3.26 (s, 3H), 3.05 (s, 1H), 2.26-0.80 (m, 21H), 0.91 (s, 3H), 0.66 (s, 3H); ^{13}C NMR (100 MHz, $CDCl_3$) δ 140.4, 121.1, 94.3, 89.1, 76.6, 69.1, 61.6, 54.8, 51.2, 50.0, 42.4, 39.3, 37.5, 37.0, 36.5, 32.0, 31.4, 31.2, 28.6, 27.6, 23.2, 20.4, 19.1, 11.3.

Steroid 4. To a solution of steroid **3** (1.82 g, 4.64 mmol) in dichloromethane (50 mL) was added Dess-Martin periodinane (2.18 g, 5.15 mmol) at room temperature. After 2 h, water was added and the mixture was extracted into dichloromethane (100 mL x 2). Solvent was removed under reduced pressure and the residue was purified by flash column chromatography (silica gel eluted with 10% EtOAc in hexanes) to give steroid **4** (1.70 g, 93%): ^{1}H NMR (400 MHz, $CDCl_3$) δ 9.71 (s, 1H), 5.27 (d, *J* = 4.2 Hz, 1H), 4.60 (s, 2H), 3.75-3.68 (m, 2H), 3.37-3.23 (m, 2H), 3.29 (s, 3H), 2.56-0.83 (m, 21H), 0.94 (s, 3H), 0.67 (s, 3H); ^{13}C NMR (100 MHz, $CDCl_3$) δ 201.3, 140.6, 121.1, 94.4, 89.2, 76.6, 63.6, 54.9, 51.3, 50.0, 44.0, 42.6, 39.4, 37.6, 37.1, 36.5, 31.5, 31.3, 28.7, 27.7, 23.2, 20.5, 19.2, 11.2.

Steroid 5. To a suspension of magnesium turnings (396 mg, 16.5 mmol) in diethyl ether (15 mL) was added 1,2-dibromethane (0.1 mL) and then 4-bromo-1-trimethylsilyl-1-butyne (2.2 g, 11 mmol) in diethyl ether (15 mL) was added, the flask was heated initially by heat gun and dropwise addition of the alkyne kept the flask warm. After 1 h, the reaction was cooled down to –10 °C and then the aldehyde (1.70 g, 4.36 mmol) in diethyl ether (15 mL) was added. After 1 h at –10 °C, the mixture was warmed up to room temperature for 30 min. Aqueous NH_4Cl (5 mL) was added and the mixture was filtered through Celite and washed with diethyl ether (150 mL), solvent was removed under reduced pressure and the residue was redissolved in dichloromethane (40 mL). Dess-Martin periodinane (2.12 g, 5.0 mmol) was added at room temperature. After 2 h, water was added and the product was extracted into dichloromethane (100 mL x 2). Solvent was removed under reduced pressure and the residue was purified by flash column chromatography (silica gel eluted with 10% EtOAc in hexanes) to give steroid **5** (1.23 g, 55%, 2 steps): ^{1}H NMR (400 MHz, $CDCl_3$) δ 5.34 (s, 1H), 4.68 (s, 2H), 3.73-3.64 (m, 2H), 3.44-3.38 (m, 2H), 3.36 (s, 3H), 3.30 (t, *J* = 8.2 Hz, 1H), 2.73-0.74 (m, 24H), 1.01 (s, 3H), 0.73 (s, 3H), 0.13 (s, 9H); ^{13}C NMR (100 MHz, $CDCl_3$) δ 207.6, 140.8, 121.4, 105.8, 94.7, 89.4, 84.9, 76.7, 65.1, 55.1, 51.5, 50.2, 43.3, 42.8, 42.4, 39.5, 37.8, 37.2, 36.7, 31.8, 31.5, 28.9, 27.8, 23.4, 20.7, 19.4, 14.2, 11.5, 0.04 (3 x C).

Steroid 6. To a suspension of steroid **5** (1.23 g, 2.39 mmol) in methanol (30 ml) was added ammonia in methanol (7.0 M, 30 mL, 210 mmol) at room temperature. After 3 h, hydroxylamine-*O*-sulfonic acid (297 mg, 2.63 mmol) in methanol (10 mL) was added. After 16 h, methanol was removed under reduced pressure, water (20 mL) was added to the residue the product was extracted into

dichloromethane (100 mL x 2). The organic layers were dried, filtered and the solvents removed. The residue was redissolved in methanol (30 mL), Et_3N (1.0 mL) was added, followed by I_2 in methanol until a brown color persisted. Aqueous $Na_2S_2O_3$ (50 mL) was added and the reaction mixture was extracted into dichloromethane (100 mL x 2) and washed with brine (50 mL x 2). The organic layers were dried, filtered and the solvents removed. The residue was purified by flash column chromatography (silica gel eluted with 10% EtOAc in hexanes) to give steroid **6** (365 mg, 29% in 2 steps): ^{1}H NMR (400 MHz, $CDCl_3$) δ 5.35 (d, J = 3.6 Hz, 1H), 4.68 (s, 2H), 3.43-3.39 (m, 1H), 3.37 (s, 3H), 3.28-3.21 (m, 3H), 2.36-0.88 (m, 25H), 1.02 (s, 3H), 0.78 (s, 3H), 0.14 (s, 9H); ^{13}C NMR (100 MHz, $CDCl_3$) δ 140.8, 121.4, 105.7, 94.6, 89.3, 85.2, 76.7, 64.4, 55.1, 51.5, 50.3, 42.7, 39.5, 37.7, 37.2, 36.7, 33.5, 33.2, 31.7, 31.5, 28.9, 27.8, 27.1, 23.4, 20.7, 19.4, 14.7, 11.5, -0.01 (3 x C).

Steroid 7. Steroid **7** without the trimethylsilyl group (120 mg, 11%) was also isolated along with steroid **6**. Steroid **7** was directly converted to steroid **9** by removal of the methoxymethoxy group using the procedure given below for the preparation of steroid **8** from steroid **6**. Steroid **7** had: ^{1}H NMR (400 MHz, $CDCl_3$) δ 5.35 (d, J = 3.6 Hz, 1H), 4.68 (s, 2H), 3.44-3.38 (m, 1H), 3.36 (s, 3H), 3.28-3.22 (m, 3H), 2.36-0.89 (m, 26H), 1.01 (s, 3H), 0.78 (s, 3H); ^{13}C NMR (100 MHz, $CDCl_3$) δ 140.8, 121.4, 94.6, 89.4, 83.0, 76.7, 68.9, 64.3, 55.1, 51.5, 50.3, 42.7, 39.5, 37.7, 37.2, 36.7, 33.5, 32.9, 31.7, 31.5, 28.9, 27.8, 27.0, 23.4, 20.7, 19.4, 13.3, 11.6.

Steroid 8. To a solution of steroid **6** (365 mg, 0.69 mmol) in methanol (30 mL) was slowly added acetyl chloride (3 mL) at room temperature. After 2 h, water was added and the product was extracted into dichloromethane (100 mL x 2). The organic layers were dried, filtered and concentrated. The residue was purified by flash column chromatography (25% of EtOAc in hexanes) to give the steroid **8** (330 mg, 99%): ^{1}H NMR ($CDCl_3$) δ 5.33 (d, J = 4.7 Hz, 1H), 3.53-3.45 (m, 1H), 3.26-3.19 (m, 3H), 2.29-0.89 (m, 26H), 0.99 (s, 3H), 0.76 (s, 3H), 0.12 (s, 9H); ^{13}C NMR (100 MHz, $CDCl_3$) δ 140.8, 121.2, 105.7, 89.3, 85.2, 71.5, 64.3, 51.4, 50.2, 42.7, 42.2, 37.7, 37.2, 36.5, 33.4, 33.1, 31.6, 31.5, 31.4, 27.7, 27.0, 23.4, 20.7, 19.3, 14.6, 11.5, -0.04 (3 x C).

Steroid 9 (MQ-182). To a solution of steroid **8** (330 mg, 0.68 mmol) in THF (10 mL) was added TBAF (1.0 M in THF, 2.0 mL, 2.0 mmol) at room temperature. After 2 h, THF was removed under reduced pressure and the residue was purified by flash column chromatography (25% of EtOAc in hexanes) to give the product (259 mg, 93%): $[\alpha]_D^{20}$ –39.0 (c = 0.20, $CHCl_3$); ^{1}H NMR ($CDCl_3$) δ 5.32 (d, J = 4.7 Hz, 1H), 3.52-3.45 (m, 1H), 3.26-3.20 (m, 3H), 2.29-0.89 (m, 27H), 0.99 (s, 3H), 0.76 (s, 3H); ^{13}C NMR (100 MHz, $CDCl_3$) δ 140.8, 121.3, 89.4, 82.9, 71.6, 68.9, 64.3, 51.5, 50.2, 42.7, 42.2, 37.7, 27.2, 36.5, 33.5, 32.9, 31.7, 31.6, 31.4, 27.8, 27.0, 23.4, 20.7, 19.4, 13.3, 11.5; IR ν_{max} 3266, 1565, 1442 cm^{-1}.
